# Foreign Summary

**Published:** 1839-07-13

**Authors:** 


### FOREIGN SUMMARY.

*Clinical Lecture on an obscure Case of Peritonitis.* By Robert Carswell, M. D.—The next case is that of Ann Kimber, who died on the 28th of last month. This patient was admitted on the 19th with symptoms of rather an anomalous character, the principal of which, however, were referrible to rheumatism, and with which she had been affected two months before. The following is a general outline of her case.

Four days previous to her admission she felt feverish; had pain in the bowels, headach, and pains in the joints. When admitted she complained of pain in all the larger joints; less when warm; no swelling, heat, nor redness; she had very little power in either hand; also pain and tenderness on pressure in many parts of the abdomen, but chiefly in the right hypochondriac region, in the epigastrium, and over the kidneys. The pains were relieved by lying on her face. The skin was dry and harsh; there was some headach; tongue whitish; no appetite; considerable thirst; bowels confined; urine small in quantity, dark in colour, and depositing a sediment; respiration natural; heart normal; pulse ninety-eight, full and hard; suppressed menstruation for the last two months.

There being no redness or swelling of the affected joints, and the febrile symptoms not being urgent, antiphlogistic treatment was not considered necessary; the extract of colchicum was ordered, one grain, three times a day, with eight grains of Dover's powder, and two grains of calomel, at night.

On the 21st, two days afterwards, she complained more of her head, and had been occasionally delirious during the night, in consequence of which the head was shaved, and eight ounces of blood taken from the nape of the neck. After the use of these means she passed the two succeeding nights better, but as there was still some headach, six leeches were ordered to be applied behind the ears on each side; and as the rheumatic pains were still present, the colchicum was continued and increased from one to two grains.

The pain and tenderness first felt by the patient at her admission, and referred to several parts of the abdomen, but chiefly in the right hypochondrium, epigastrium, and region of the kidneys, were not made the subject of complaint during the four following days. However, when pressure was employed the patient did complain of some pain, and generally in different parts of the abdomen; but it was always, up to this period, of very doubtful character. I considered it to be either entirely of a rheumatic character, or partly of this nature, and increased by the occasional accumulation of flatus.

On the 25th, that is, on the fifth day of admission of the patient, the case presented an entirely new feature. There occurred considerable pain in the lower part of the abdomen, during the morning of that day, and in the evening there was a profuse discharge of blood and coagula from the vagina. On the following day the discharge continued, and also the pain in the region of the uterus; and, on examination per vaginam, the orifice of the uterus was found much dilated, and a coagulum hanging from it into the vagina. Some of the discharged clots were examined, and were found to consist of coagulated blood and fibrinous matter, having a somewhat membranous appearance, but presenting no evidence of the presence of an ovum. However, it was supposed that the patient was pregnant when admitted, and that the discharge was the consequence of abortion; and the supposition acquired confirmation from the circumstance that the menses had been suppressed for the last two months; that the patient had previously given birth to a child, although unmarried, and that she had been heard to allude to pregnancy at the time she was delirious.

With this opinion, as to the nature of the sanguineous discharge from the uterus, no means were employed to arrest it until the following day. The face and surface of the body were then very pale, the countenance anxious, and the pulse one hundred, and small. She was then ordered ʒij. of the sulphate of magnesia, with M x. of dilute sulphuric acid, in ʒx. of water, twice a day. This was on the 26th, the discharge of blood still continuing. On the 27th she complained of constant pain in the abdomen, greatly increased by the slightest pressure; there was more anxiety of countenance; greater pallor; pulse very weak, and one hundred and forty; tympanitic state of the abdomen. Examined again per vaginam, the os uteri was still dilated, and filled with coagula; the uterus itself was not very tender.

With the view chiefly of relieving the flatulent distention of the abdomen and pain, a turpentine injection was administered, two minims of creosote to be taken immediately, and repeated if necessary, and half a grain of the muriate of morphia. When seen again in the evening, the severity of the pain continued unabated, and, although it had become probable that the pain was now dependent on peritonitis, no active antiphlogistic measures could be adopted. The patient, indeed, was already reduced to a state of great prostration; she was nearly exsanguinous. She was, therefore, ordered five grains of blue pill every four hours, and a large blister to be applied to the hypogastrium.

On the moning of the following day there was some relief from the pain; there was less flatulency; but the prostration was increasing; the pulse as high as one hundred and fifty, and extremely weak. She was now evidently sinking, and although wine was administered every two hours, she became suddenly worse, and died at half past four in the evening.

The history of this case presents itself to our consideration under several important points of view, but chiefly as regards the occurrence of the peritonitis and the nature of the discharge from the uterus. The existence of the peritonitis was certainly not suspected till two days previous to the death of the patient; and it becomes an important inquiry how far the abdominal pains previously complained of were dependent upon inflammation of the peritoneum. It was on the *fourth* day previous to the death of the patient that these pains became considerable, and it was also on this day that the discharge from the uterus, or abortion, occurred; consequently we naturally attributed their aggravation to this circumstance; I am not disposed, however, to believe that was the case; that is to say, that the peritonitis was the consequence of the previously diseased state of the uterus. For the disease of the uterus was not of such a kind as to affect, by contiguity, the peritoneum; and if it had done so, we should have found, at the examination of the body, appearances which would have shown such a mode of extension. We should have found the peritonitis, if not confined to the peritoneum covering of the fundus of the uterus and pelvic viscera, at least more severe there than in other parts of the abdominal cavity, which was not the case. The inflammation occupied the greater part of the visceral peritoneum, but chiefly of the intestines and stomach. On the contrary, I am disposed to believe, for these reasons, that the peritonitis occurred as an idiopathic disease, and very probably after the admission of the patient, from exposure to cold, probably when, in a state of delirium, she got out of her bed during the night. With this view of the case we may explain, what is by no means a rare occurrence, the supervention of abortion, as a consequence of the peritonitis. I have already stated that it was only on the morning of the 24th, the day on which the discharge from the uterus took place, that the pain became severe in the lower part of the abdomen. Whether this was the *commencement* of the peritonitis, or an *aggravation* of it, we have no means of determining; but on the evening of that day a profuse sanguineous discharge, as I have described, took place, and continued, more or less, so as to occasion general pallor and great prostration up to the day of the patient's death. No other means of treatment could, under these circumstances, have been adopted to arrest the progress of the peritoneal inflammation, than those which were had recourse to, viz., counter-irritation, and the speedy introduction of mercury into the system.

With regard to the next question, viz., the nature of the contents of the uterus, I have already said that the examination of the discharge afforded no evidence of the existence of pregnancy, no trace of a fœtus, nor of the membranes of the ovum.

I have already mentioned the condition in which the os and cervix uteri were found before death; the suspicious language of the patient, and the fact of menstruation having been suppressed for a period of two months, and of the patient having already had an illegitimate child.

In order to throw additional light on this subject, let us see in what state the uterus and ovaries were found after death. The uterus was much increased in bulk, and the right ovary considerably enlarged; the cavity, but especially the neck of the uterus, was considerably dilated, and contained a quantity of dark, coagulated blood, a cylindrical portion of which projected through the os uteri into the vagina. The os uteri was also dilated sufficiently to admit the point of the middle finger, and the surface of this part and of the cervix was of a deep-red colour. A rather soft, shaggy, deep-red substance, about the size of the point of the finger, resembling the tissue of the placenta, was attached to the surface of the fundus of the uterus, near the opening of the right fallopian tube. The right ovary, (the larger of the two, although the left was larger than natural,) was of a somewhat ovate form, thicker at one extremity than the other, and presented, as well as the left, slight traces of peritoneal inflammation. When the right was cut into it was found to contain two serous cysts, one of which might have contained a small pea, the other a very small cherry. An *orange-yellow-coloured* substance, of an oval form, about three-eighths of an inch in diameter, was situated in the substance of the ovary, *immediately beneath* the peritoneal covering, and towards the *enlarged* extremity of the ovary. The central portion of this substance presented very faint traces only of a cavity, and no appearance of a cicatrix. In two points of the substance, but deep-seated, of the same ovary, were observed orange-red-coloured substances of a lesser size, and of an irregular shape. In the left ovary the latter appearances were also seen, but none of the orange-yellow-coloured substance found in the right ovary.

From these appearances observed in the uterus and ovaries, and from the previous facts of the case already stated, I have no hesitation in saying that this woman must have been in a state of pregnancy when admitted into the hospital. Independently of the suspicious circumstance of the patient's having already given birth to an illegitimate child, we have before us changes in the uterus and ovaries corresponding with the period of the supposed pregnancy, and some of which are almost universally admitted to be the result of impregnation alone. The increased size of the uterus, may, certainly, depend on a variety of causes; but in this case it was accompanied by the presence of a substance resembling the remains of the placenta, and attached to the fundus of the uterus, near the opening of the right fallopian tube, that corresponding with the right ovary, and through which the ovum must have passed. But the most important of these changes were those observed in this, the right ovary. It was larger than the left; somewhat protuberant at one extremity, as happens after impregnation; and contained a peculiar substance situated where the impregnated ovum always must be, viz., beneath the peritoneal covering, before its escape. This substance I believe to be what is called the *corpus luteum*, although not so well marked an example of it as I have seen on several occasions. It presented the colour, glandular-looking structure, and general form of this body. In order to have presented a perfect resemblance of the *corpus luteum* it should have had a central cavity, or, what is a still more marked character, a stellated cicatrix, which is the result of the union of the perforated membranes through which the ovum makes its escape. However, notwithstanding the absence or imperfect traces of these, I conceive that the other appearances were sufficient to identify the existence of a *corpus luteum* in this case, and, consequently, of conception.

For a luminous and accurate review of all the circumstances connected with this important question, I beg to refer you to the work of Dr. W. F. Montgomery, on the "Signs and Symptoms of Pregnancy," &c.

I shall only further observe, that although we have, in this case, evidence of conception, we have none that it was fruitful, or that a fœtus was developed. We had no traces of the membranes of an ovum, far less of a fœtus, in the substances discharged during life, although the first of these may not have been submitted to our examination. With regard to the orange-red coloured substances found in several points both of the right and left ovaries, it is of importance to be remembered that they are not an uncommon occurrence, and the consequence of circumscribed hæmorrhage. Here, as in hæmorrhage of the brain, for example, the serous part of the effused blood is absorbed, and, after a certain time, the red-colouring matter becomes more and more of an orange tint; the fibrine becomes consolidated and organised, and may, subsequently, undergo a variety of transformations. In the present case the effused blood was in the fibrinous state, and undergoing the change of colour mentioned.—*Lancet.*

---

*Case of Pregnancy of the Fallopian Tube.* By Dr. Bamberger, of Berlin.—Philippine N., æt. thirty-four, had spent ten years of a former marriage childless, and had already passed from three to four years of a second marriage without having a child. On the evening of the 4th of March, whilst playing at cards with a few friends, she suddenly left the room, and was found a few minutes afterwards sitting upon the sofa in another room, complaining of faintness and nausea, which she ascribed to a pain in the abdomen, which she had begun to feel about half an hour before. The tongue was coated, the pulse scarcely perceptible, and the face deadly pale. The pain in the abdomen was not very violent, and was accompanied by a bearing down in the rectum. The patient was disposed to ascribe her complaints to the delay of the menses, which should have appeared six or seven days before; the bowels were constipated, but the other functions were in a normal state. Vomiting ensued in about a quarter of an hour, and removed almost all the uneasiness, although the face still continued very pale, and the pulse was small, wiry, and quick. At eleven she went to bed, assuring her friends that she felt perfectly well. She could not, however, fall asleep, and the vomiting returned several times through the night. At four in the morning she complained of pain in the abdomen, especially in the gastric region, and of a feeling of tightness reaching from the stomach to the neck, which rendered every movement painful. The abdomen felt somewhat tense, and was painful on pressure, but the gastric region was more sensible than the parts below the umbilicus. The pulse was quick and wiry, and the cold perspiration stood on the forehead and limbs. As there had been no motion of the bowels, castor oil was prescribed, and an injection administered; cupping-glasses were at the same time applied to the abdomen. In consequence of these measures the tenseness of the abdomen was somewhat diminished; and another injection succeeded in producing motion of the bowels, and a quantity of hard scyballa were passed. The condition of the patient, nevertheless, did not improve; small quantities of ice were now given to check the vomiting, and leeches were applied to the abdomen. She slept some hours during the night, but the vomiting still continued. At twelve she became very low, and one fainting fit succeeded upon another, till she expired about half past seven in the evening.

On examining the body, a large quantity of blood was found in the cavity of the abdomen, and the left fallopian tube was burst about its middle by an ovum about the size of a pigeon's egg. The fœtus was not found in the ovum, and it had probably escaped into the cavity of the abdomen.

Dr. Bamberger was at first inclined to ascribe the symptoms to obstruction of the bowels; but he was afterwards obliged to assume the presence of internal hæmorrhage, although he could not determine its cause. He appears to have suspected that extra-uterine pregnancy might have been the cause of the phenomena; but not to have attached much value to this supposition, as the pain was less acute than he would have suspected in pregnancy of the tube, and the characteristic cry, as described by Heim, (see Br. and For. Med. Rev., Vol. IV. p. 493,) was wanting.—*Brit. and For. Med. Rev., from Casper's Wochenshrift*, No. 39, 1838.

